# Extensive regulation of nicotinate phosphoribosyltransferase (NAPRT) expression in human tissues and tumors

**DOI:** 10.18632/oncotarget.6538

**Published:** 2015-12-09

**Authors:** Sara Duarte-Pereira, Isabel Pereira-Castro, Sarah S. Silva, Mariana Gonçalves Correia, Célia Neto, Luís Teixeira da Costa, António Amorim, Raquel M. Silva

**Affiliations:** ^1^ IPATIMUP - Institute of Molecular Pathology and Immunology of the University of Porto, Porto, Portugal; ^2^ ICAAM - Instituto de Ciências Agrárias e Ambientais Mediterrânicas, University of Évora, Évora, Portugal; ^3^ Faculty of Sciences, University of Porto, Porto, Portugal; ^4^ Instituto de Investigação e Inovação em Saúde, Universidade do Porto, Porto, Portugal; ^5^ Institute for Biomedicine - iBiMED & IEETA, University of Aveiro, Aveiro, Portugal; ^6^ Gene Regulation Group, i3S/IBMC - Instituto de Investigação e Inovação em Saúde/Instituto de Biologia Molecular e Celular, Universidade do Porto, Porto, Portugal

**Keywords:** human NAD salvage, NAPRT, NAMPT, alternative transcripts, anti-cancer therapies

## Abstract

Nicotinamide adenine dinucleotide (NAD) is a cofactor in redox reactions and a substrate for NAD-consuming enzymes, such as PARPs and sirtuins. As cancer cells have increased NAD requirements, the main NAD salvage enzymes in humans, nicotinamide phosphoribosyltransferase (NAMPT) and nicotinate phosphoribosyltransferase (NAPRT), are involved in the development of novel anti-cancer therapies. Knowledge of the expression patterns of both genes in tissues and tumors is critical for the use of nicotinic acid (NA) as cytoprotective in therapies using NAMPT inhibitors. Herein, we provide a comprehensive study of *NAPRT* and *NAMPT* expression across human tissues and tumor cell lines. We show that both genes are widely expressed under normal conditions and describe the occurrence of novel *NAPRT* transcripts. Also, we explore some of the *NAPRT* gene expression mechanisms. Our findings underline that the efficiency of NA in treatments with NAMPT inhibitors is dependent on the knowledge of the expression profiles and regulation of both *NAMPT* and *NAPRT*.

## INTRODUCTION

Nicotinamide adenine dinucleotide (NAD) is a coenzyme in oxidation-reduction reactions that release energy in the form of ATP. Additionally, it is a substrate for NAD-consuming enzymes such as poly(ADP-ribose) polymerases (PARPs) and sirtuins [1].

NAD-consuming enzymes are important in several cell functions, from gene silencing to DNA repair, cell signaling and cell survival, which has raised interest in the biosynthesis of NAD. In humans, NAD can be synthesized from different precursors, namely tryptophan in the *de novo* pathway and nicotinamide (Nam), nicotinamide riboside (NR) and nicotinic acid (NA) through distinct salvage pathways [2, 3].

NAD synthesis from Nam is catalyzed by nicotinamide phosphoribosyltransferase (NAMPT), which is the first and rate-limiting enzyme of this pathway, and has been associated with several diseases, including inflammation, metabolic disorders and cancer [4]. Cancer cells have high energetic requirements and a high rate of NAD turnover, as well as increased demands for DNA repair activity [4-6]. As NAMPT overexpression has been reported in various tumor types, such as colorectal, ovarian, gastric, prostate, lung and glioblastoma [7-12], NAMPT inhibitors have been developed as potential anticancer agents [13-17].

In this context, nicotinate phosphoribosyltransferase (*NAPRT*) is also being studied [17-20]. NAPRT is responsible for the first step of the conversion of NA to NAD. Although Nam is the main NAD salvageable precursor in human cells, most likely because this molecule is itself produced in NAD-consuming reactions [2], NA is more efficient in increasing NAD levels, thus, some tissues could preferentially use the NAD salvage pathway from NA [3, 21]. Lack of *NAPRT* expression in some tumors, such as neuroblastoma or glioblastoma [17], and lymphomas [22], places *NAPRT* as a biomarker for the use of NA as a chemoprotectant agent in the treatment with NAMPT inhibitors [19]. In *NAPRT*-negative tumors, inhibiting NAMPT provides a novel synthetic lethal therapeutic approach by inducing metabolic stress, while normal cells are rescued by NA via activation of the *NAPRT* pathway [17, 18, 23].

In order to predict the usefulness of NA-therapeutics, knowledge of the expression patterns of both human *NAPRT* and *NAMPT* is mandatory [6], but few studies have focused on the characterization of human *NAPRT* [24]. Most data concerning *NAPRT* expression refers to mouse and rat tissues [21] or human tumors [17, 19], but a thorough survey of *NAPRT* expression patterns in humans is missing. Here, we describe our results of a global study of *NAPRT* and *NAMPT* expression across human tissues and tumor cell lines. We show that both genes are widely expressed and that *NAPRT* is subject to extensive gene regulation, and discuss the implications of these findings for NA-protective therapeutics.

## RESULTS

### *NAPRT* and *NAMPT* expression

**Figure 1:**
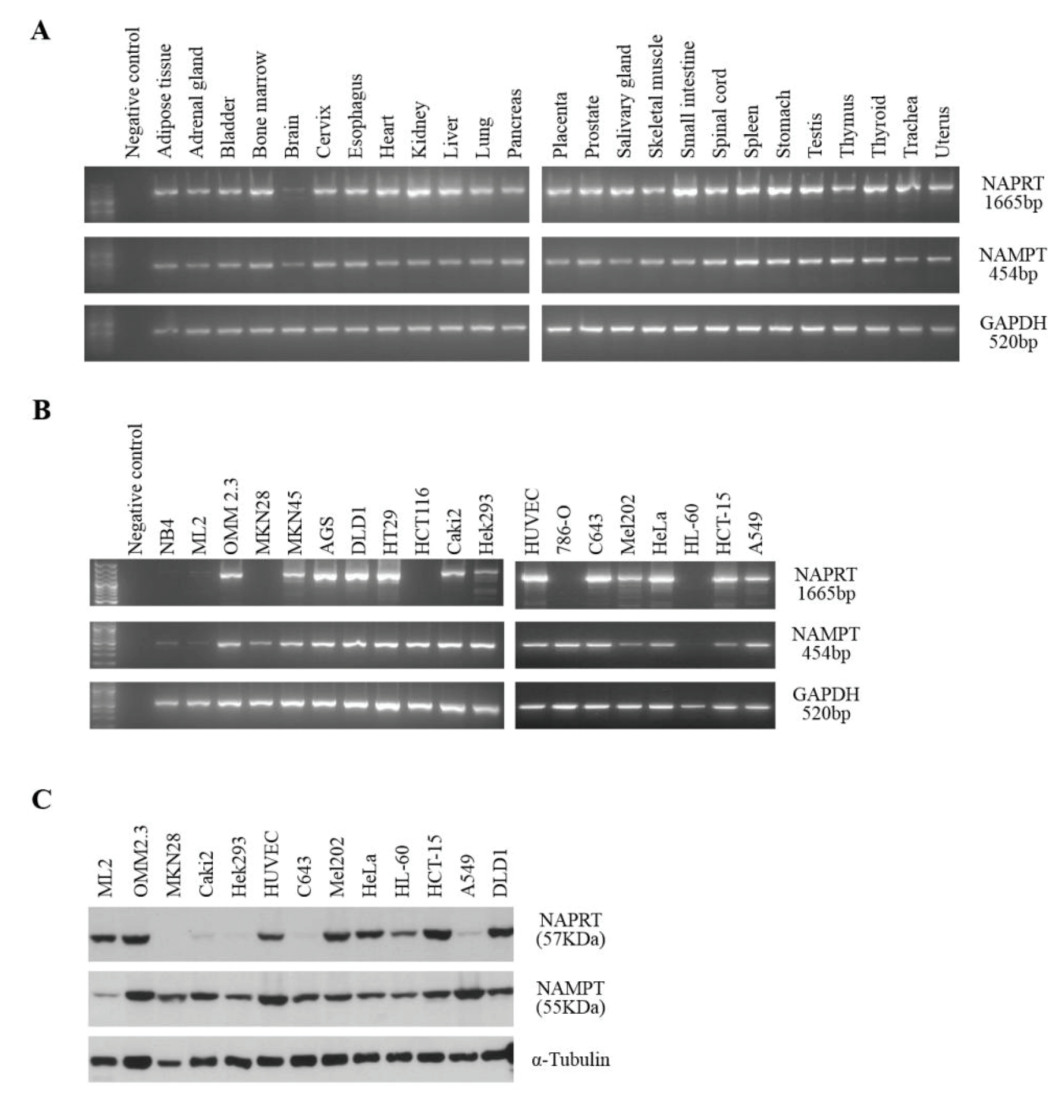
NAPRT and NAMPT expression in normal human tissues and in tumor cell lines **A**. RT-PCR analysis of RNA from several human tissues shows that both genes are widely expressed in all tissues tested. Similar results were obtained from independent cDNA synthesis and with tissues from different sources. *GAPDH* is included as a control. **B**. RT-PCR analysis of RNA from human tumor cell lines shows some variability of both *NAPRT* and *NAMPT* among the tumor types tested. *GAPDH* is included as a control. **C**. Western-blot analysis reveals distinct patterns of NAPRT and NAMPT protein expression. α-tubulin is shown as a loading control.

The initial step in this study was to evaluate *NAPRT* and *NAMPT* expression in a set of normal human tissues. RT-PCR results showed a widespread expression for both genes (Figure 1A). Curiously, the brain tissue appeared to express less *NAMPT* and *NAPRT.* Bioinformatics analyses using expressed sequence tag (EST) data available in the UniGene database [25] from NCBI showed that *NAMPT* is broadly expressed, whereas several tissues had no reported expression of *NAPRT* (Supplementary Figure 1). Data from the Human Protein Atlas database [26] showed that *NAPRT* and *NAMPT* mRNA were detected in all tissues, however, protein levels were highly diverse and not detected in several cases (Supplementary Figure 1).

To assess *NAPRT* and *NAMPT* expression in tumors, we studied a set of cell lines, which included renal, thyroid, cervix, lung, gastric and colorectal carcinomas, uveal melanoma, and leukemia, by RT-PCR and western blot (Figure 1B and 1C). We observed that *NAMPT* is expressed in all tumor types tested, although the leukemia cell lines (NB4, ML2 and HL-60) showed weaker expression (Figure 1B). Also, we found that the *NAPRT* gene is differentially expressed between cell lines (Figure 1B), with a marked decrease in expression in carcinoma cell lines 786-O (renal), MKN28 (gastric), HCT116 (colorectal) and in all leukemia cell lines tested (HL-60, NB4 and ML2). The western blot analysis confirmed that among the cell lines tested, Caki-2 and HEK293 (renal), C643 (thyroid) and A549 (lung) had weak NAPRT expression, and in MKN28 (gastric) the protein was undetectable (Figure 1C). NAMPT protein was detected in all cell lines analyzed.

As NAPRT-negative tumors can be targeted by NA rescue with NAMPT inhibitors, we further explored gene expression regulatory mechanisms as described below.

### *NAPRT* gene expression regulation

**Figure 2:**
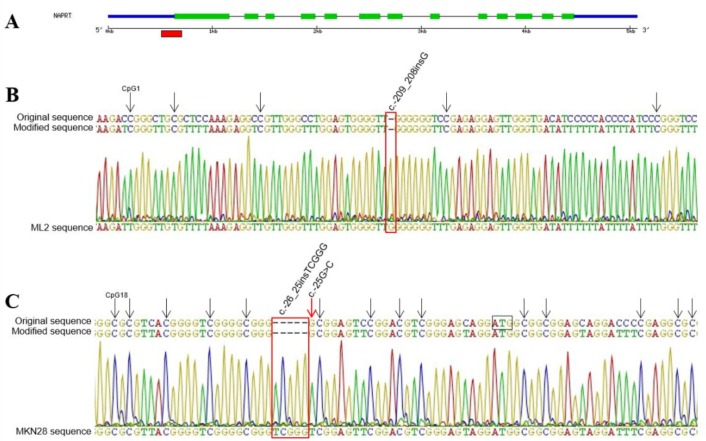
Methylation status of *NAPRT* promoter region **A**. Schematic representation of the gene structure, with the red box marking the sequenced region within the CpG island. **B**. Section of the unmethylated ML2 cell line sequencing electropherogram, showing that all cytosines including those in CpG sites (arrows) were converted to thyimines. **C**. Sequence showing the region surrounding the start codon (ATG) in MKN28 cell line, where all cytosines in GpG sites indicated by arrows are methylated including the additional CpG resultant from rs146163552 insertion. All mutations are indicated in red.

Given the absence of *NAPRT* expression in some tumor cell lines, we considered the occurrence of DNA cytosine methylation in the promoter region as previously suggested [19]. We analyzed a fragment, which included a total of 37 CpG sites, in selected cell lines with different mRNA expression (A549, C643, 786-O, HT29, HCT116, MKN28, AGS, HL60, ML2 and NB4) and observed that only the MKN28 cell line presented all cytosines methylated (Figure 2), which explains its silencing.

Also, we screened the promoter region for mutations in 13 tumor cell lines, by sequencing 436bp upstream the start codon. In this region, we detected seven variants (Supplementary Table 1). Two of these (rs146163552 and rs2305495) were found only in cell line MKN28, and one previously undescribed substitution (c.-256C>A) appeared only in HL60 cell line. The remaining variants were found in almost all analyzed cell lines.

**Figure 3:**
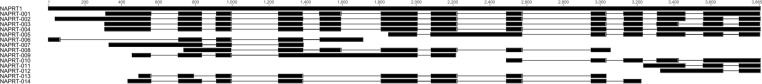
Alternative *NAPRT* transcripts predicted in Ensembl Genome Browser Alignment of the predicted *NAPRT* transcripts from Ensembl, showing the differences to the reference sequence. The transcript *NAPRT-002* corresponds to the reference sequence (NM_145201.5). Transcripts *NAPRT-003 and -004* are protein coding. *NAPRT1-003* has a deletion of 39 base pairs corresponding to 13 amino acids in exon 11, while the remaining sequence is maintained. *NAPRT1-004* retains part of intron 11 resulting in a premature STOP codon. Transcripts *NAPRT-001* and *-010,* classified as nonstop mediated decay, retain intron 12 and the consequent alteration of the reading frame leads to the readthrough of the STOP codon. Transcripts *NAPRT-013* and *-014,* classified as nonsense mediated decay, lack exon 5, yet their sequence is incomplete. The remaining transcripts have several retained introns and no complete sequence is described.

To evaluate the prevalence of alternative transcripts in *NAPRT*, we focused on the sequence and tissue of origin of *NAPRT* ESTs deposited in UniGene (Supplementary Figure 2). Considering that known alternative protein coding *NAPRT* transcripts differ in the 3′ end sequence (Figure 3), we selected a small set of tissues, namely brain, liver, skeletal muscle and small intestine to be analyzed by 3′-RACE. This choice took into account both the expression levels and the diversity of transcripts of the *NAPRT* gene. We observed the existence of two fragments in the same tissue (Supplementary Figure 3A), although the shorter transcript was undetectable in the liver. Both transcripts share an identical 3′ UTR sequence and lack a recognizable polyadenylation signal (Supplementary Figure 3B).

Sequencing analysis of both fragments revealed that the most abundant one (A) is equivalent to the reference *NAPRT* transcript (*NAPRT-002*, Figure 3), and the shorter sequence (S) is a novel transcript that lacks exons 11 and 12. Two ESTs lacking exons 11 and 12 were detected in testis tissue and in squamous cell carcinoma of the skin (UniGene database, EST ID: GenBank entries HY025556.1 and BG676963.1, respectively), yet, a complete sequence is missing.

### Novel *NAPRT* transcripts

**Figure 4:**
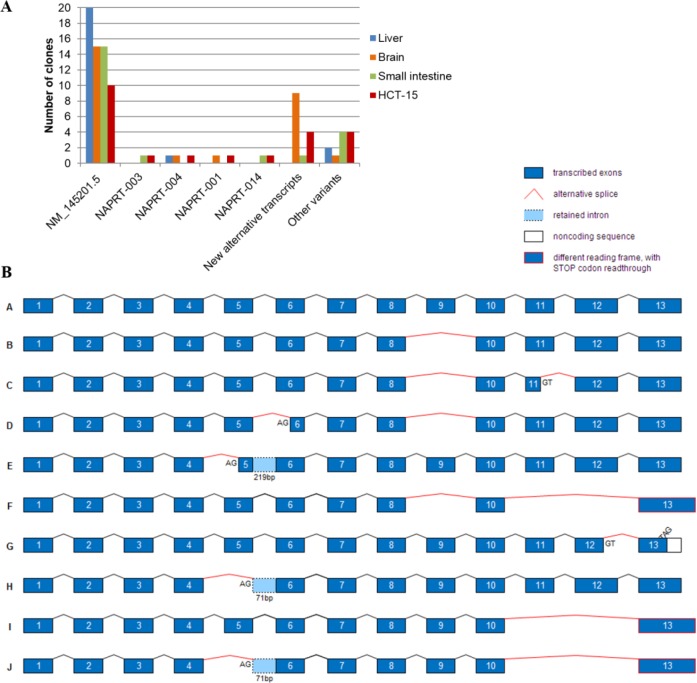
Novel alternative *NAPRT* transcripts Alternative spliced sequences were considered when donor and acceptor splice sites were identified, while a total of 10 variants presenting several exon deletions with no recognizable splice sites are referred to as other variants. **A**. Tissue distribution of *NAPRT* transcripts detected by cloning of the full-length cDNA from normal human tissues (liver, brain, small intestine) and HCT-15 tumor cell line. **B**. Schematic representation of *NAPRT* alternative transcript sequences (not in scale). Alternative splice sites (AG or GT) other than intron location, as well as premature STOP codons (TAG) are identified in each sequence. Sequence A, *NAPRT* RefSeq NM_145201.5; sequences B-E, found in brain; sequence F, found in small intestine; sequences G-J, found in HCT-15 cell line.

To obtain the full-length sequence of the novel *NAPRT* alternatively spliced transcripts, we amplified the whole coding sequence of three representative human normal tissues, liver, brain and small intestine, and a colorectal carcinoma cell line (HCT-15) and cloned the resulting RT-PCR products. The 94 clones sequenced revealed high diversity in *NAPRT* sequence (Figure 4A). In addition to *NAPRT-003*, *-004, -001*, *-014 and -005* (Figure 3), 9 novel alternative transcripts were detected and are schematically represented in Figure 4B.

The most frequent of these new transcripts was found exclusively in the brain and lacks exon 9 (Figure 4B, sequence B). The removal of this exon could suppress NAPRT enzyme activity, as critical amino acids are located in exon 9 [24, 27].

The skipping of exons 11 and 12 (Figure 4B, sequences F, I and J) was identified in small intestine and in HCT-15 cell line. This results in STOP codon readthrough and may tag the mRNA to a mechanism of nonstop mediated decay, similarly to *NAPRT-001 and -010* transcripts (Figure 3).

Exon 5 skipping appeared more than once, as well as the total or partial retention of intron 5 (Figure 4B, sequences E, H, J). These may decrease NAPRT activity, due to the location of several amino acids that bind ATP, PRPP and nicotinic acid [24, 27].

Skipping of exons 5, 9 and 11-12 were detected alone or in combination. Other alternative splicing events occurred in the middle of exons, leading to partial deletion of exon 5, exon 6, exon 11 or exon 12 (Figure 4B).

### *NAPRT* and *NAMPT* mutations

We have previously identified *NAPRT* variants in predicted splicing sites [28], thus, we further screened several tumor cell lines for mutations. Selected regions from *NAPRT* and *NAMPT* were amplified and sequenced to detect the presence of potentially damaging mutations, such as nonsense or frameshift in coding sequences. Most alterations found were silent or intronic variants already described in existing databases, and detailed information of these results is depicted in Supplementary Table 2.

Of note, the *NAPRT* rs2015562 and rs2305496 variants that are predicted as splicing or gene expression modulators were found in the MKN28 cell line [28, 29]. In *NAMPT*, one missense variant was identified in the pulmonary tumor A549 cell line (g.23531A>G, Lys339Glu). To evaluate the effect of this alteration, we predicted the structure of the NAMPT protein with the Lys339Glu replacement and observed that all polar contacts of this residue are lost in the 3D model (Supplementary Figure 4). Although the Lys339Glu substitution was predicted as benign by PolyPhen (http://genetics.bwh.harvard.edu/pph2/), this and several other *NAMPT* missense mutations have been found in lung and upper aerodigestive tract tumors, as reported in The Catalogue of Somatic Mutations in Cancer (COSMIC) [30].

Globally, these variants should not be disregarded without further data, as some are predicted to alter splicing sites or gene expression levels, and the effect of coding silent mutations in protein expression or function is increasingly recognized in the context of human disease as well [31-33].

## DISCUSSION

NAD salvage enzymes are essential in the regulation of cellular metabolism and are associated with human disease [34]. Among them, *NAMPT* has been extensively characterized and is a prospective therapeutic target in human cancer [13, 16, 35, 36]. *NAPRT*, on the other hand, has been scarcely studied, despite being a potential biomarker for the use of nicotinic acid in treatments with NAMPT inhibitors [6, 19, 20]. This strategy will be most beneficial in patients with tumors that overexpress NAMPT and/or downregulate NAPRT, while expressing normal NAPRT levels in healthy cells, which drove us to perform a global study on the expression patterns of both *NAMPT* and *NAPRT* genes in normal human tissues and tumor cell lines.

We observed that both genes are ubiquitously expressed at the mRNA level in all normal human tissues tested. While definitive conclusions on the expression of both genes would benefit from a quantitative approach, it is interesting to note the weaker expression of *NAPRT* and *NAMPT* that was observed in the brain, both in mRNA (Figure 1A) and protein expression (not shown). These results are in agreement with previous findings that the main routes to NAD in neurons are the *de novo* biosynthesis from tryptophan and the NAD salvage from nicotinamide riboside [3, 37, 38].

In tumor cell lines, we found that the *NAPRT* gene is more widely expressed than expected, at the mRNA level, considering that several cell lines are negative for the protein (Figure 1B and 1C). Even while expressing *NAPRT* transcripts, thyroid and lung carcinoma cell lines do not show protein expression, suggesting that these are good targets for NA therapy [39-41]. On the other hand, in some cell lines such as leukemia ML-2 and HL-60, the full-length transcript was barely detected, although expression of the NAPRT protein was found. These results imply that both transcriptional and post-transcriptional mechanisms control the expression of the *NAPRT* gene in tumor cell lines, thus, we searched for the presence of alternative transcripts and sequence alterations both in coding and regulatory regions.

Our results support the presence of more than one *NAPRT* transcript in most tissues (Supplementary Figure 3 and data not shown), all with identical 3′ end sequence. This indicates that *NAPRT* is subjected to alternative splicing but not to alternative polyadenylation. Additionally, the transcripts have no recognizable polyadenylation signal within the 10-30 nucleotide interval upstream of the cleavage site. As 20 to 30% of human genes yield transcripts with no detectable polyadenylation signal in their 3′ UTRs [42], this seems to be the case of *NAPRT*. We also described the sequences of novel *NAPRT* transcripts resulting from alternative splicing (Figure 4). These have different functional consequences, for example, transcripts with premature STOP codons or STOP codon readthrough may be eliminated by nonsense or nonstop mediated decay. In others, the loss of amino acids involved in enzyme activity or in establishing the dimer interface may result in NAPRT inactivation (Supplementary Table 3 and Supplementary Figure 5).

Some *NAPRT* transcripts appear to be tissue-specific. In our study, brain showed the highest number of alternative transcripts, which is supported by previous observations in brain cells, showing over 40% prevalence of alternative splicing [43, 44]. Considering that the reference transcript is also less expressed in brain, a regulatory role for these alternative *NAPRT* transcripts described here should be further considered.

As RNA binding proteins (RBPs) mediate post-transcriptional regulation, we searched for putative RBPs binding sites in the *NAPRT* sequence using the DoRiNA database (available at http://dorina.mdc-berlin.de) [45]. We found six predicted binding sites for Argonaute proteins, which are known transcriptional silencers as part of the RNA-induced silencing complex [46], and have been recently linked to alternative splicing [47]. In *NAPRT* sequence, two of the Argonaute binding sites are localized in regions lost in two alternative transcripts found in brain (Figure 4B, sequences C and E). Also, and although most of the splicing factors are ubiquitously expressed, some have been described as tissue-specific [48]. Among them, is the neuro-oncologic ventral antigen 1 (NOVA-1) protein, a brain specific RBP implicated in neurological disorders [49]. NOVA proteins control alternative splicing events by specifically recognizing pre-mRNA containing YCAY motifs [50]. If the YCAY motif is located within the exon, it would be removed from the mature mRNA [51]. Considering that *NAPRT* has two putative *NOVA1* binding sites in exon 9, namely, the UCAU and UCAC motifs in positions 17 and 47 (according to SFmap web server available at http://sfmap.technion.ac.il/ [52]), NOVA binding could be a possible mechanism for the alternative splicing of *NAPRT* exon 9 in the brain.

Beyond alternative transcripts, we identified seven variants within the 500bp upstream the *NAPRT* start codon. We searched for potential human transcription factor binding sites (TFBS) applying the online tool TFSEARCH (Searching Transcription Factor Binding Sites, http://www.rwcp.or.jp/papia/), which is based on the TRANSFAC database [53], to evaluate the effect of such alterations on the prediction of TFBS. We observed loss of cAMP Response Element-Binding protein binding site (CREB) for rs3214817, and gain of Acute Myeloid Leukemia 1 protein binding site (AML1a) for rs896949, which were found in most of the cell lines studied. The TCGGG insertion (rs146163552), which was found only in the *NAPRT*-negative cell line MKN28, was predicted to result in the loss of one Specificity Protein 1 (Sp1) binding site and decrease of the score for another Sp1 binding site. Sp1 binds with high affinity to GC-rich motifs [54], which are present in the *NAPRT* promoter.

Additionally, because DNA methylation was recently established as a mechanism to suppress *NAPRT* gene expression [19], we evaluated the methylation status of the *NAPRT* promoter region in tumor cell lines. The methylation detected in the MKN28 cell line promoter sequence supports the hypothesis of epigenetic inactivation of *NAPRT*, since this was the only case for which no mRNA nor protein expression was found. In the other cell lines with weak *NAPRT* expression no methylation was found, strengthening the idea of tissue/cancer type specificity of expression and regulation mechanisms.

Overall, we demonstrate that *NAMPT* and *NAPRT* are ubiquitously expressed in normal human tissues, and we have detected novel *NAPRT* transcripts, in normal tissues and in tumor cell lines. Furthermore, we identified several modulators of the *NAPRT* gene expression, from mutations in transcription factor binding sites, to promoter methylation and alternative splicing. These mechanisms should be further investigated to understand the influence of the *NAPRT* gene in NAD metabolism in different tissues and disease conditions. In particular, evidence on how is *NAPRT* expression regulated in tumor cells could spark developments to expand the use of NA cytoprotective therapies in treatments using NAMPT inhibitors.

## MATERIALS AND METHODS

### Bioinformatics analysis

Expressed sequence tags (ESTs) of human *NAPRT* (NM_145201.4-5) and *NAMPT* (NM_005746.2) genes were extracted from the UniGene database [25] at NCBI (http://www.ncbi.nlm.nih.gov/unigene/), as counts per million transcripts. A heat map was created with the collected EST data displayed as Log_2_ transcripts per million using the web interface matrix2png v1.2.1 [55], following a previously described methodology [56]. Gene and protein expression data from both NAPRT and NAMPT was retrieved from Human Protein Atlas database, version 13, Nov2014 (www.proteinatlas.org) [26], and was classified into four categories of expression according to database annotations.

UniGene sequences representing the *NAPRT* gene were assembled and aligned against the mRNA RefSeq NM_145201.4-5 using the MUSCLE software [57] implemented in Geneious v5.5.6 [58]. A total of 196 sequences was analyzed and matched to one of the *NAPRT* transcripts described in Ensembl release 80, May 2015 (http://www.ensembl.org/) [59]. Sixty ESTs too small (< 300bp) to match any of the 14 described transcripts or those identified in our work, or containing different alterations were excluded from this approach. Additionally, the tissue distribution of all analyzed ESTs was determined.

### Samples and cell lines

Total RNAs from human tissues consisted in the FirstChoice® Human Total RNA Survey Panel, from Ambion (Austin, TX, USA), the Human Total RNA Master Panel II from Clontech (Mountain View, CA, USA), and Pancreas and Adrenal Gland Total RNA from AMSBio (Oxon, UK). Tumor cell lines from ATCC - American Type Culture Collection (MD, USA) - included renal (786-O and Caki-2), cervix (HeLa), colorectal (HCT-15, DLD1, HCT116 and HT-29), gastric (AGS, MKN28 and MKN45) and lung (A549) carcinomas, leukemia (HL-60, NB4 and ML2), and embryonic cell lines HUVEC and HEK293. Thyroid carcinoma cell line C643 was obtained from Prof. Marc Mareel (Laboratory of Experimental Cancerology, Ghent University Hospital, Ghent, Belgium). Uveal melanoma cell lines Mel202 and OMM2.3 were established by Dr. B.R. Ksander (Schepens Eye Research Institute, Boston, USA) [60, 61] and were kindly provided by Dr. M. J. Jager (LUMC, Leiden, The Netherlands).

### DNA, RNA and protein extraction

DNA, total RNA and protein were extracted from the cell lines using the Illustra TriplePrep Kit (GE Healthcare, Buckinghamshire, UK), according to the manufacturer's instructions. In all cases, genomic DNA was removed from RNA preparations using RNase-free DNase I, (Fermentas, Thermo Fisher Scientific Inc., Waltham, MA, USA) as follows: one microgram of RNA was incubated at 37°C for 30 min with 1 unit of DNase I, using the 1x reaction buffer containing MgCl_2_. Then, 1 μL of 50 mM EDTA was added and incubated at 65°C for 10 min to inactivate the enzyme. The prepared RNA was used as a template for reverse transcriptase. Protein concentrations were determined by the Quick Start Bradford Assay (Bio-Rad Laboratories, Hercules, CA, USA).

### Polymerase chain reaction (PCR) and reverse transcription PCR (RT-PCR)

Complementary DNA (cDNA) was synthesized from 1 μg of RNA using the RETROscrip® First Strand Synthesis Kit (Ambion) with oligo-dT primers according to the manufacturer's instructions.

Amplification reactions from 1 μL of either DNA or the synthesized cDNA were prepared in a 10 μL final volume reaction, using 5 μL of HotStarTaq® Master Mix Kit (Qiagen, Germantown, MD, USA). Each primer final concentration was 0.2 μM and Q solution (Qiagen) was included in the reaction (10%). The complete list of primers, regions amplified and PCR conditions is shown in Supplementary Table 4. Expression of the glyceraldehyde 3-phosphate dehydrogenase gene (*GAPDH*, reference transcript NM_002046) was used as a control in RT-PCR reactions.

All amplification products were visualized on 1.5% agarose gels and confirmed by sequencing. Gel image acquisition was processed with Quantity-One 1-D Analysis Software Version 4.6.8 (Bio-Rad, Hercules, CA, USA).

### 3′ Rapid amplification of cDNA ends (RACE) analysis

3′ RACE cDNA was synthesized using the SMARTer™ RACE cDNA Amplification Kit (Clontech), as previously described [62]. The 3′ RACE PCR was performed with a reaction mixture containing 12.5 μL of 2x QIAGEN multiplex PCR master mix (Qiagen), 8.5 μL of water, 2.5 μL of 10x Universal Primer A Mix (Clontech), 0.5 μL of the gene specific primer (10 μM) and 1 μL of the 3′ RACE cDNA in a final volume of 25 μL. Amplification conditions were carried out as indicated in the SMARTer™ RACE Kit protocol. Following the first PCR reaction, nested PCR was performed using 1 μL of the 3′ RACE PCR product in 12.5 μL of 2x QIAGEN multiplex PCR master mix (Qiagen), 9.5 μL of water, 1 μL of Nested Universal Primer (Clontech), and 1 μL (10 μM) of the nested gene specific primer (Supplementary Table 4) in a final volume of 25 μL.

3′ RACE products were resolved by 1.5% agarose gel electrophoresis, which image was acquired with Quantity-One Software (Bio-Rad), each DNA band was extracted from gel, purified and sequenced with the gene specific primers.

### Sequencing analysis

PCR products were purified with ExoSAP-IT (USB Corporation, Santa Clara, CA, USA) by incubation at 37°C for 15 min, followed by enzyme inactivation for 15 min at 85°C, according to the manufacturer's instructions. The resulting purified fragments were sequenced using the ABI Big Dye Terminator Cycle Sequencing Ready Reaction kit v3.1 (Applied Biosystems, Life Technologies Corporation, Carlsbad, CA, USA) and analyzed in an ABI PRISM 3130xl (Applied Biosystems).

### Transcript cloning

*NAPRT* full-length transcripts from the liver, brain, small intestine tissues and from HCT-15 cell line were cloned in a TA-cloning plasmid (pUK-TA, in-house developed). A total of 94 clones were selected, plasmid DNA was extracted and sequenced as described above using the TA-F and TA-R primers [63]. The DNA sequence of the different clones was aligned against the *NAPRT* reference mRNA sequence as described above.

### Western-blot analysis

Total protein extracts from cell lines (15 μg each) were separated in a 10% SDS-PAGE and transferred into a nitrocellulose Amersham Hybond-ECL membrane (GE Healthcare). Rabbit polyclonal antibodies anti-NAPRT (HPA023739) from Sigma (St. Louis, MO, USA) and anti-PBEF (H-300) from Santa Cruz Biotechnology (Santa Cruz, CA, USA) were used at a 1:1000 dilution in 1% non-fat milk PBS Tween-20. Mouse monoclonal anti-α-tubulin antibody (Sigma) was used at a dilution of 1:40000 to assess protein loading. Goat anti-rabbit and goat anti-mouse horseradish peroxidase-conjugated secondary antibodies (Santa Cruz Biotechnology) were used at 1:5000, and developed with the ECL-Plus detection kit (GE Healthcare).

### DNA bisulfite treatment and sequencing

The presence of promoter CpG islands in the *NAPRT* sequence region ranging from 1000 bp upstream to 500 bp downstream the transcription start site was evaluated using the online CpG Island Searcher software (http://cpgislands.usc.edu/) with default settings.

DNA samples from selected cell lines were bisulfite modified with the EpiTect® Fast Bisulfite Conversion kit (Qiagen), according to manufacturer's instructions. Primer sequences specifically designed to amplify a 356bp fragment (from −281 to +75) within the CpG island in modified DNA are indicated in Supplementary Table 2. PCR products were visualized in 9% polyacrylamide gels by silver staining and were subjected to direct sequencing as described above.

### Protein modeling and structure visualization

The human NAMPT (PDB id: 3DKJ) [64] and NAPRT (PDB id: 4YUB) [27] structures were used as a template in MODELLER [65] to build structural models containing mutations. The structures were visualized using Pymol v1.1r1 software [66].

## SUPPLEMENTARY MATERIAL FIGURES AND TABLES


