# Supplementary material for: Extensive regulation of nicotinate phosphoribosyltransferase (NAPRT) expression in human tissues and tumors
Source: Oncotarget. 2015 Dec 9;7(2):1973–83. doi: 10.18632/oncotarget.6538 (PMC4811510; doi:10.18632/oncotarget.6538)
Supplement: Supplementary file 1 [file oncotarget-07-1973-s001.pdf]

Extensive regulation of nicotinate phosphoribosyltransferase (NAPRT) expression in human tissues and tumors

Supplementary Material

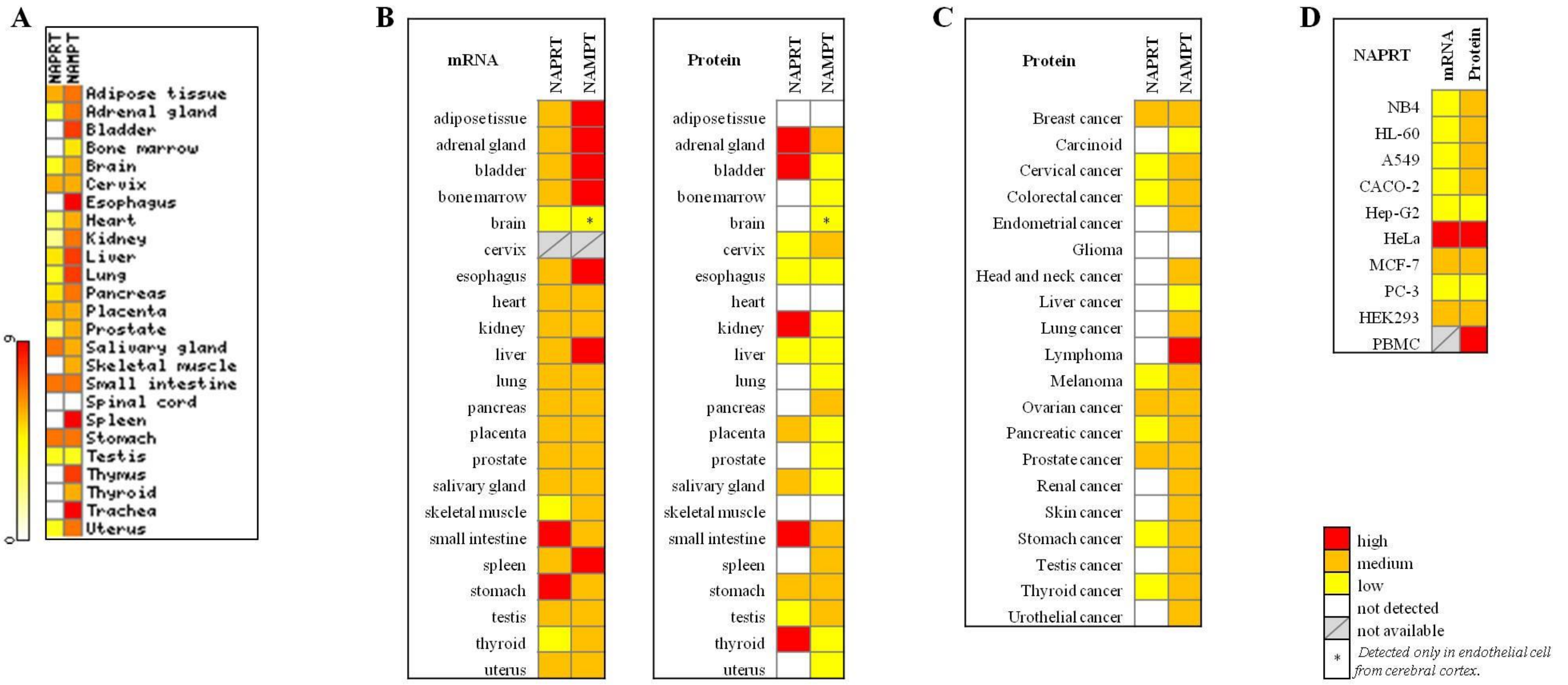

Supplementary Figure 1: Expression profiles of human NAPRT and NAMPT from Unigene (A) and Human Protein Atlas (B, C, and D) databases. A. EST profiles of human *NAPRT* and *NAMPT* retrieved from UniGene are represented as log2 of EST counts by color coding. **B.** Expression profiles of normal tissues, both at the mRNA and protein level. **C.** Protein expression from cancer tissues. **D.** NAPRT mRNA and protein expression in selected cell lines. The mRNA expression data is derived from deep sequencing of RNA and the protein expression data is derived from antibody-based protein profiling using immunohistochemistry. Presented data refers to information available online on 1st June 2015 at www.proteinatlas.org and was categorized in high, medium, low or not detected level of expression according to database annotations.

A

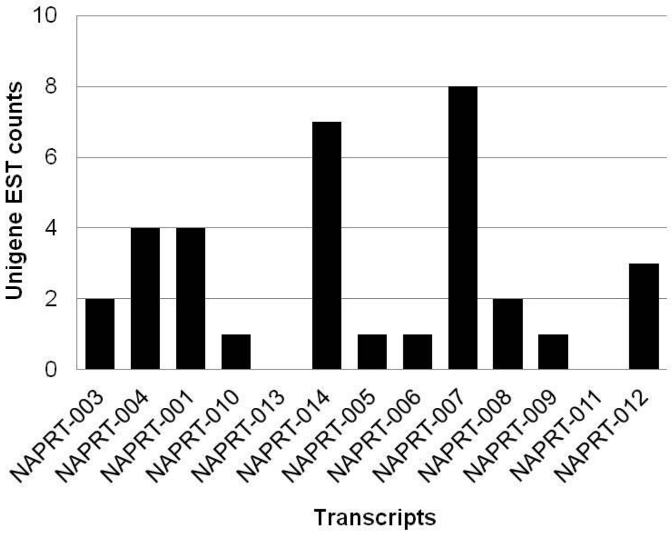

B

Transcripts (Ensembl)		Tissues
**protein coding**	NAPRT-003	testis, uncaractherized
	NAPRT-004	brain, testis, intestine, pancreas
**nonsense/non-stop mediated decay**	NAPRT-001	intestine, liver, uncaractherized
	NAPRT-010	conective tissue
	NAPRT-013	-
	NAPRT-014	testis
**retained intron**	NAPRT-005	uncaractherized
	NAPRT-006	testis
	NAPRT-007	placenta, ovary, testis, intestine
	NAPRT-008	brain, pancreas
	NAPRT-009	testis
	NAPRT-011	-
	NAPRT-012	liver, uncaractherized
**Total alternative ESTs :**		34
**Total ESTs analyzed:**		196

Supplementary Figure 2: Alternative *NAPRT* transcripts predicted in Ensembl Genome Browser and detected in UniGene databases. A. Number of counts of *NAPRT* alternative transcripts represented in the EST database UniGene. **B.** Tissues in which the different transcripts were found, excluding the reference sequence.

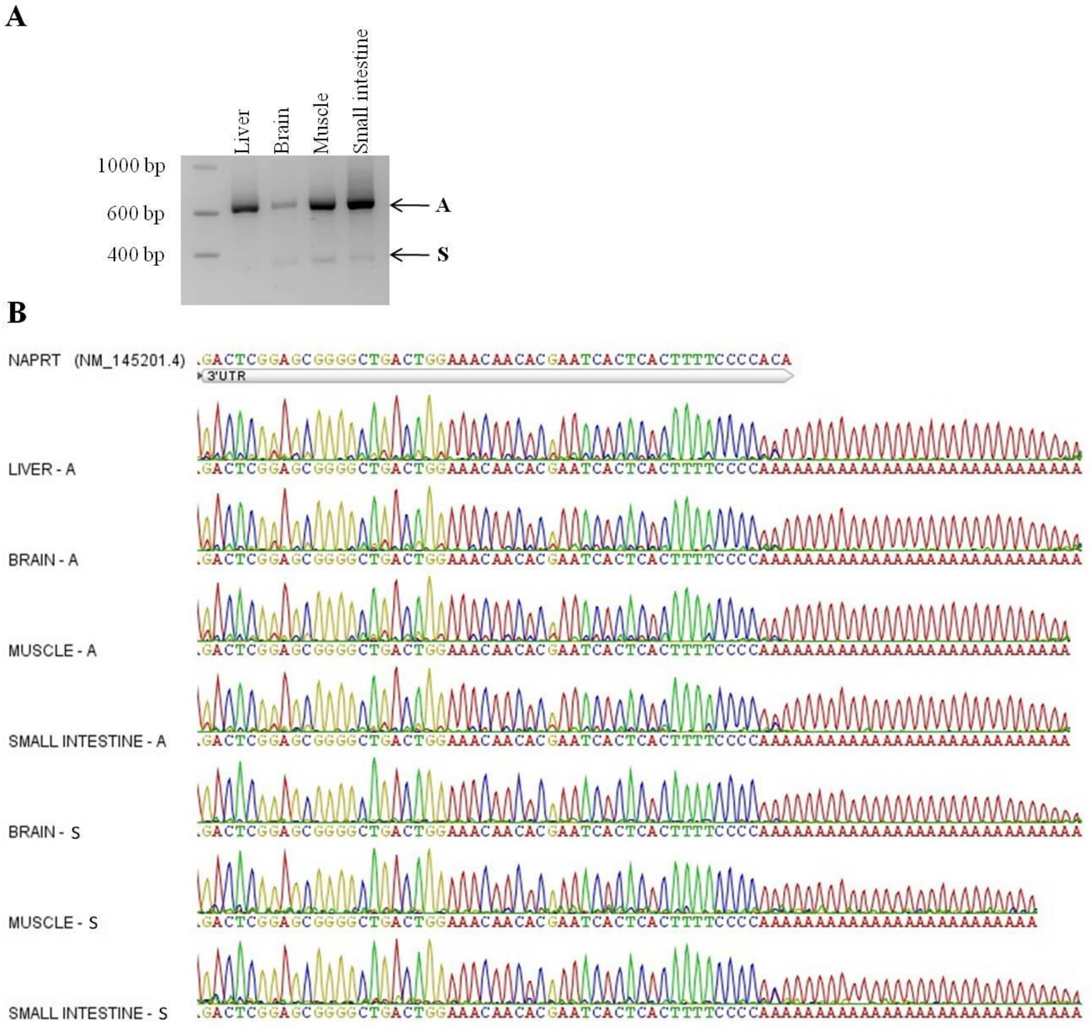

Supplementary Figure 3: *NAPRT* transcripts characterization by 3'-RACE. A. RACE analysis of *NAPRT* mRNA 3' ends revealed the presence of two transcripts in the same tissue, the reference sequence (A) and the smaller fragment (S). **B.** Sequencing of the two different transcripts in brain, muscle and small intestine showed that the 3' end is similar in the two transcripts and no conventional polyadenylation signal was detectable. The smaller fragment (S) lacks exons 11 and 12, originating the difference in size between the two transcripts.

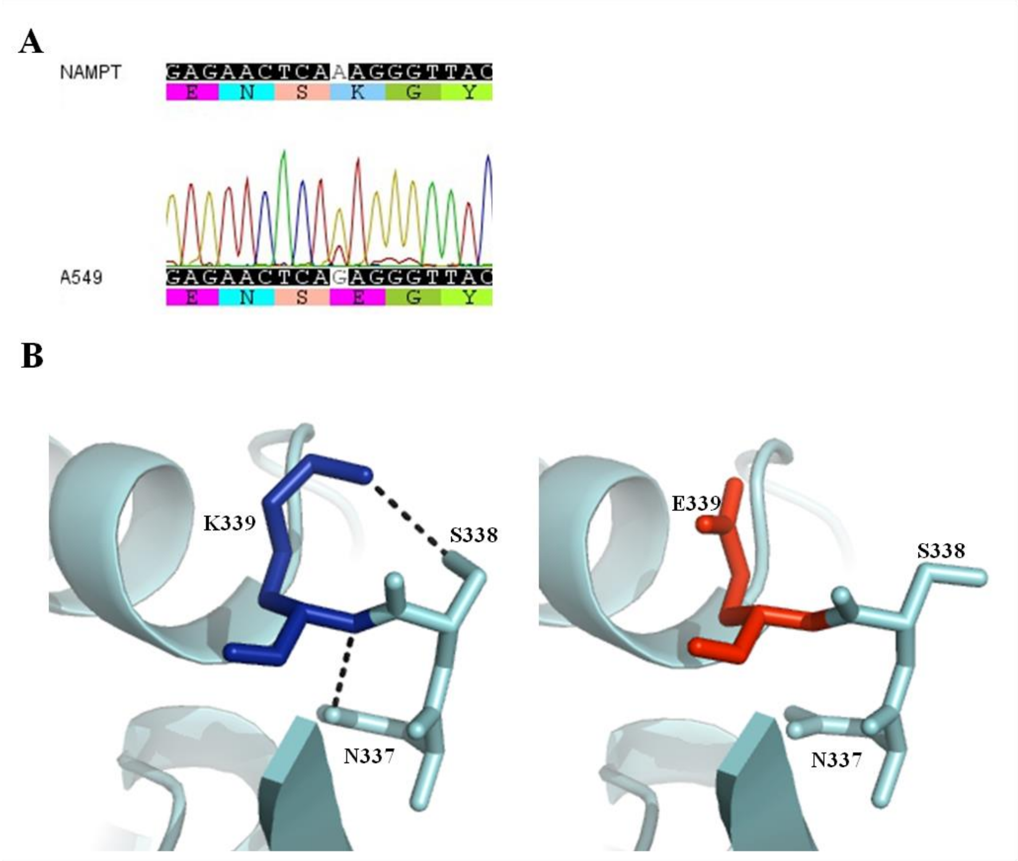

Supplementary Figure 4: NAMPT missense mutation Lys339Glu. A. Electropherogram showing g.23531A>G replacement in heterozygosity that leads to Lys339Glu substitution in NAMPT protein. **B.** Structural model representing Lys339 (blue), which establishes polar contacts with Asn337 and Ser338. In the model with Glu339 (red) those polar contacts are lost.

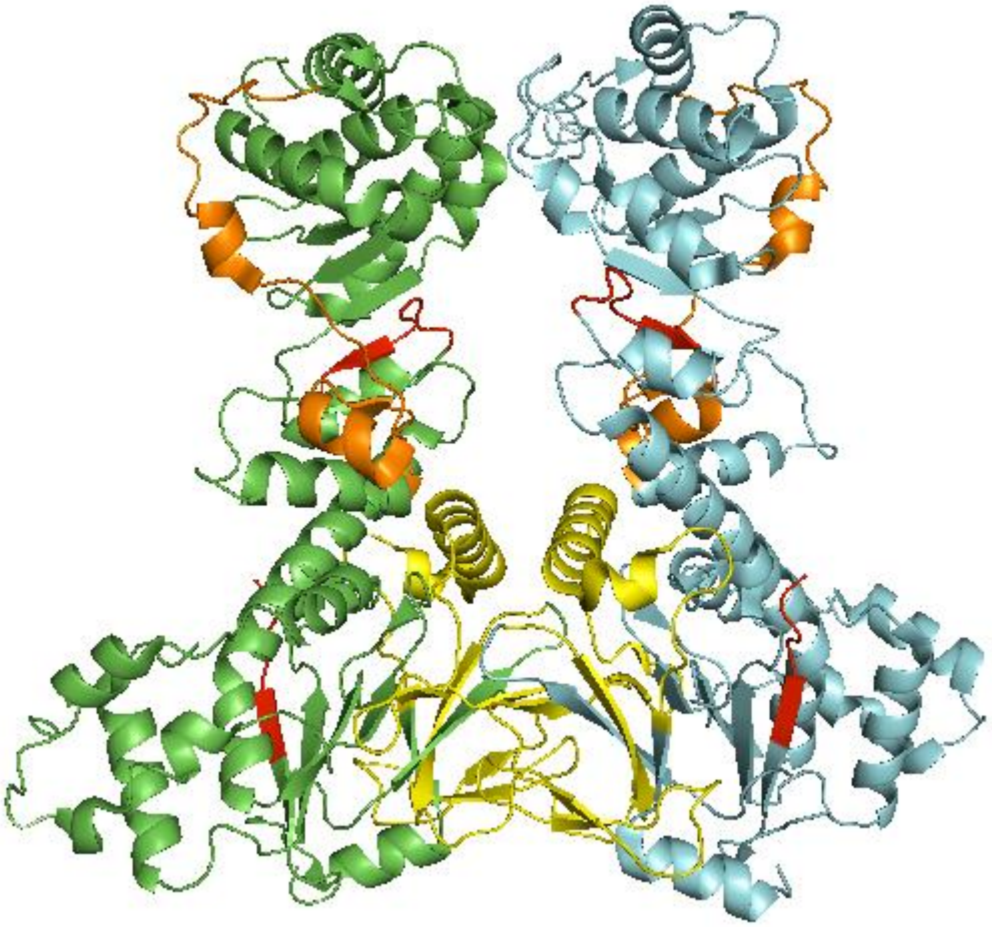

Supplementary Figure 5: Crystal structure of human Nicotinic Acid Phosphoribosyltransferase (PDB ID: 4YUB) [27]. Exons 5 (orange) and 9 (red) are partly located in the active center and exons 11 and 12 (yellow) are located in the dimer interface. The alternative transcripts missing these exons will most probably result in enzyme inactivation.

Supplementary Table 1. Allele frequencies of human *NAPRT* promoter genetic variants. Seven alterations were detected in *NAPRT* putative promoter region. Predicted Impact of each mutation was retrievedfrom RegulomeDB (http://regulomedb.org/).

**Ref ID**	**Variant type**	**Nucleotide change**	786	C643	HUVEC	NB4	HL60	A549	HEK293	AGS	CAKI2	HCT116	HT29	MKN28	ML2	RegulomeDB
rs6996126	5´near gene	c.-340G>T	homozygous	homozygous	homozygous	homozygous	homozygous	homozygous	homozygous	homozygous	homozygous	homozygous	homozygous	homozygous	homozygous	4 - minimal binding evidence
-	5´near gene	c.-256C>A	wild-type	wild-type	wild-type	wild-type	homozygous	wild-type	wild-type	wild-type	wild-type	wild-type	wild-type	wild-type	wild-type	-
rs3214817	5´near gene	c.-209_208insG	homozygous	heterozygous	heterozygous	heterozygous	wild-type	homozygous	homozygous	homozygous	homozygous	homozygous	heterozygous	wild-type	heterozygous	2b - likely to affect binding
rs896949	5´near gene	c.-87G>C	homozygous	heterozygous	heterozygous	heterozygous	wild-type	homozygous	homozygous	homozygous	homozygous	homozygous	heterozygous	homozygous	heterozygous	4 - minimal binding evidence
rs4475500	5´near gene	c.-35T>C	homozygous	homozygous	homozygous	homozygous	homozygous	homozygous	homozygous	homozygous	homozygous	homozygous	homozygous	homozygous	homozygous	2b - likely to affect binding
rs146163552	5'UTR	c.-26_25insTCGGG	wild-type	wild-type	wild-type	wild-type	wild-type	wild-type	wild-type	wild-type	wild-type	wild-type	wild-type	homozygous	wild-type	2b - likely to affect binding
rs2305495	5'UTR	c.-25G>C	wild-type	wild-type	wild-type	wild-type	wild-type	wild-type	wild-type	wild-type	wild-type	wild-type	wild-type	homozygous	wild-type	2b - likely to affect binding

(dark)	homozygous for the variant
(grey)	heterozygous for the variant
(white)	wild-type

Supplementary Table 2. Allele frequencies of human *NAMPT* and *NAPRT* genetic variants. Partial sequencing of the genes revealed two intronic and one silent variants in *NAMPT*. Also, one missense mutation was identified in A549 cell line. In *NAPRT*, five intronic and two silent variants were found. Predicted Impact of each mutation was retrievedfrom RegulomeDB (http://regulomedb.org/).

Gene	Ref ID	Variant type	Exon / Intron	Nucleotide change	Amino acid change	786	C643	MEL202	DLD1	HUVEC	HELA	NB4	HL60	HCT15	A549	HEK293	CAKI2	MKN28	ML2	RegulomeDB
NAMPT	rs28454100	Intronic	intron 4	g.13195T>G	-						▒									6 - minimal binding evidence
	rs34056375	Intronic	intron 4	g.13422G>A	-		▒			■			■		▒					6 - minimal binding evidence
	rs2302559	Silent	exon 7	g.21735A>G	Ser301Ser		■			■	■	■	■							5 - minimal binding evidence
	-	Missense	exon 8	g.23531A>G	Lys339Glu										▒					-
NAPRT	rs2015562	Intronic	intron 1	g.257C>A	-													■		4 - minimal binding evidence
	rs896953	Intronic	intron 1	g.325T>C	-	▒	▒					▒			■		■	■	■	3a - less likely to affect binding
	rs896954	Silent	exon 2	g.468C>T	Ala98Ala		▒			▒		▒	■							4 - minimal binding evidence
	rs2305496	Intronic	intron 2	g.565G>T	-													■		1f - likely to affect binding and linked to expression of a gene target
	rs12678314	Intronic	intron 3	g.906T>C	-	■						▒			■				■	5 - minimal binding evidence
	-	Intronic	intron 5	g.1303delC	-	▒														-
	rs872935	Silent	exon 7	g.1803C>T	Leu305Leu	■	▒	■	■	▒	▒	▒		■	■					5 - minimal binding evidence

■	homozygous for the variant
▒	heterozygous for the variant
	wild-type

Supplementary Table 3. Functionality prediction of the protein isoforms derived from novel *NAPRT* alternative transcripts.

Tissue	Sequence	Alternative splicing events	Consequence	Known role of lost residues	Predicted effect on function
Brain	B	deletion of exon 9	loses T380, S381, K396	ATP and PRPP binding [27]	decreased/ lost activity
	C	deletion of exon 9	loses T380, S381, K396	ATP and PRPP binding [27]	decreased/ lost activity
		partial deletion of exon 11	deletion of 13 aa	(alteration in the protein coding NAPRT-003 transcript)	
	D	partial deletion of exon 6	deletion of 56 aa		
		deletion of exon 9	loses T380, S381, K396	ATP and PRPP binding [27]	decreased/ lost activity
	E	partial deletion of exon 5	loses S195, N196, T210, A212, F215, H213, S214	NA binding, ATP activation, PRPP stabilization [24,27]	decreased/ lost activity
		retained intron 5	insertion of 73 aa		
Intestine	F	deletion of exon 9	loses T380, S381, K396	ATP and PRPP binding [27]	decreased/ lost activity
		deletion of exons 11 and 12	deletion of 88 aa (from 431 to 518)		loses interface for dimerization
			altered reading frame, STOP readthrough		
HCT-15	G	partial deletion of exon 12	premature stop condon		
	H	deletion of exon 5	loses S195, N196, T210, A212, F215, H213, S214	NA binding, ATP activation, PRPP stabilization [24,27]	decreased/ lost activity
		partially retained intron 5	insertion of 23 aa		
	I	deletion of exons 11 and 12	deletion of 88 aa (from 431 to 518)	location of the dimer interface [27]	loses interface for dimerization
			altered reading frame, STOP readthrough		
	J	deletion of exon 5	loses S195, N196, T210, A212, F215, H213, S214	NA binding, ATP activation, PRPP stabilization [24,27]	decreased/ lost activity
		partially retained intron 5	insertion of 23 aa		
		deletion of exons 11 and 12	deletion of 88 aa (from 431 to 518)		loses interface for dimerization
			altered reading frame, STOP readthrough		

Supplementary Table 4. Oligonucleotide sequences used for amplification of *NAMPT* and *NAPRT* genes.

Gene	Sample type	Location	Primer forward (5’-3’)	Primer reverse (5’-3’)	Annealing temperature (ºC)	Product size (bp)
NAMPT	DNA	Exon 2	CTTCAAGCCTTTTCTGTTGTG	CTTAGAACATCAAACACACACC	60	424
		Exon 4	AGCACGTGGCAGCATTAAAC	GATGAGGAAATTGAAGCCTGG	58	502
		Exon 7	CATAACAGCTTGGGGGAAAG	CTCTCTCTGGGCTGCAACT	58	362
		Exon 8	CCCTTTAAAGTACACTGGACC	GTAAGTATGGCTTGGCTGGC	60	491
		Exon 9	CAGTCTTGGATGCTTCATTC	CTCCCTTCTTTCCTTGTTTCTG	58	402
	RT-PCR	Exon 1-4	CTCATTTTTCTCCTTCCTCGC	TCCTCTGGGAATGACAAAGC	60	454
		Exon 3-7	TCCAGGAAGCCAAAGATGTC	CTTTCCCCCAAGCTGTTATG	60	527
		Exon 6-8	GCATCTGCTCACTTGGTTAAC	CTCTAAGATAAGGTGGCAGC	60	404
		Exon 7-11	GGCACCACTAATAATCAGACC	CAAACCCCACACATCTGTAC	60	648
NAPRT	DNA	5'UTR	CTAACTAAGGCAGCCTCTGG	AGCGGCGGAAGAAGAGCTCGAAC	61	605
		Exon 1-3	ACCTCTACCAGGCCACCATG	GGTAACTGCGCTGAGACCAG	61	615
		Exon 2-5	CTGGTCTCAGCGCAGTTACC	GAGAGCAAAGGTAATGCAGC	58	1053
		Exon 5-6	CCTTTTCAGGCAGCGAGGTGCCC	CTGTAGGTGTCCAGGAGGC	60	439
		Exon 6-9	CTACAGCGTGTGGAGGTGAG	CTGTCCCTACCTTATAGACG	58	1049
		Exon 9-13	CGTGTTGTTTCCAGTCAGCC	GCTGGGTGAACAAACGACAC	60	893
	modified DNA	CGI	TGGGGGTATTTTTGGTGATTAAG	CATAATAACCTAATAAAAATCAATAAAC	55	356
	RT-PCR	Exon 1-13	GGACGTCGGGAGCAGGATG	CGTGTTGTTTCCAGTCAGCC	64	1665
		Exon 9-13	GGCATTGGCACCAGTGTGGTCACCTG	GGCATTGGCACCAGTGTGGTCACCTG	60	486
	3' RACE	Exon 9	GGCATTGGCACCAGTGTGGTCACCTG	*universal primer*	-	-
		Exon 10	GCCACGAATGAAGCTGACCGAGGAC	*nested universal primer*	-	-
GAPDH	RT-PCR		CCAGCCGAGCCACATCG	GGTCATGAGTCCTTCCACG	60	520
